# Evaluating the welfare of extensively managed sheep

**DOI:** 10.1371/journal.pone.0218603

**Published:** 2019-06-19

**Authors:** Carolina A. Munoz, Angus J. D. Campbell, Paul H. Hemsworth, Rebecca E. Doyle

**Affiliations:** 1 Animal Welfare Science Centre, The University of Melbourne, North Melbourne, VIC, Australia; 2 Faculty of Veterinary and Agricultural Science, The University of Melbourne, Werribee, VIC, Australia; Humboldt-Universitat zu Berlin, GERMANY

## Abstract

The aim of this study was to identify the main on-farm welfare issues likely to be encountered in extensive sheep farming systems. Thirty-two commercial sheep farms in Victoria, Australia were involved in this study. Of the 32 farms involved, 30 were visited twice (at mid-pregnancy and weaning), and 2 farms only once (both at weaning). In total, 62 visits were conducted and 6,200 ewes (aged 2–5 years) were examined using six animal-based indicators: body condition score (BCS), fleece condition, skin lesions, tail length, dag score and lameness. In addition, the number of ewes that needed further care (such as sick or injured sheep) was recorded and reported to the farmers. Generalised linear mixed models were conducted to investigate associations between welfare outcomes and visit, ewe breed and location, with all three, and their interactions, as fixed factors. In all instances, farm was set as a random factor to account for specific variation between farms. Overall, the welfare of the ewe flocks, based on the six indicators measured, was considered good. A total of 86.9% of the ewes were in adequate BCS (2.5–3.5), 91% had good fleece condition, 69.2% had no skin lesions, 97.1% had low dag scores, and overall lameness was 4.7%. An important and prevalent risk to welfare identified across farms was short tail length; with 85.7% of ewes having tails docked shorter than the third palpable joint. While the welfare of the flock was good, ewes in need of further care were identified at all farms. There were 185 (3.0%) cases needing further care, and the extent of welfare compromise of these animals was considered significant. Main reasons for further care were moderate/severe lameness or foot-related issues, BCS ≤ 2 and active dermatophilosis or broken wool. To our knowledge, this study constitutes the largest assessment of ewes conducted in Australia, and the findings provide valuable insight into the main welfare issues likely to be encountered in extensive sheep farming enterprises. Future studies should develop practical technologies that can assist in the detection of the welfare issues identified in this study. In addition, the thresholds identified here could be used for future comparison and sheep welfare benchmarking programs to assess farm performance and measure continuous improvements.

## Introduction

There are many types of extensive sheep production systems in the world, but some shared characteristics of extensive systems are that they rely mainly on pasture feeding as animals are managed outdoors all year round, or at least for most of their lives, with limited monitoring and human intervention [1]. These are typical production systems in the UK and the southern hemisphere, including Australia, New Zealand, South Africa, Argentina, Chile and Uruguay [1]. Extensive sheep farming systems create opportunities for sheep to live a ‘natural life’. Sheep have a free choice and control over a number of aspects of their life, including grazing, exploration, rumination, social interaction and maternal behaviours [2]. These characteristics of extensive systems fit with one of the three conceptual frameworks used to assess animal welfare, ‘natural living’ [3], and also has clear links to similar concepts in the ‘five freedoms’—*freedom to express normal behaviours* [4] and the ‘five domains’—*behavioural or interactive restriction* [5].

While the welfare of sheep is largely positive when assessed according to natural living, extensive systems create a different set of welfare issues to those that are seen in intensive (or behaviourally restrictive) production settings. There is limited research, however, investigating welfare issues in extensively managed sheep [6], and the research that does exist has largely been conducted in European countries [2,7–11]. Although there may be similarities in welfare issues between sheep farming systems in Europe and the southern hemisphere, different locations, the scale of production and weather conditions can create a different set of welfare concerns which warrants further investigation.

Sheep farms in the southern hemisphere are quite typically large-scale farms with a considerably low sheep:labour ratio when compared with European countries. In Scotland for instance, there are 2.6 million breeding ewes, with the majority in flock sizes of 500 sheep [12]. In contrast, countries such as Australia and New Zealand have 37.2 and 17.8 million breeding ewes respectively, with the majority of the Australian ewes in flocks of more than 2,800 sheep [13]. Lamb and ewe mortality, poor nutrition, intestinal parasites, lameness, provision of water and shelter, flystrike and mastitis have been identified as important welfare concerns for Australian sheep producers by survey studies [14–16] and one on-farm study [17]. While some issues likely to be encountered in these extensive systems have been identified, the extent of the issues remains unknown and needs further investigation. In this study, animal welfare assessments were conducted on 32 commercial Australian sheep farms with the aim of identifying the main welfare compromises and risks. This present study is part of a larger research investigating farmer attitudes and sheep welfare outcomes. This paper reports on the main welfare issues likely to be encountered in extensive sheep farming systems and examines opportunities to safeguard the welfare of sheep. The work reported in Munoz et al., [18] (under review by PLOS ONE) builds on this present paper and reports on the relationship between farmer attitudes, management behaviour and sheep welfare.

## Material and methods

### Farms and visits

Thirty-two commercial sheep farms, located in the high rainfall (> 600 mm) and wheat-sheep zones (300–600 mm) of Victoria, Australia were involved in the study. Farmers were recruited through advertisements in industry magazines, by engaging with local consultants and their groups, by advertising in industry conferences or through nomination by neighbours. Eligible farms had to have a self-replacing ewe flock and spring lambing and contain a minimum of 400 breeding ewes. This cut-off number was based on preliminary results from focus group discussions with Victorian farmers [19]. Wherever possible, visits were arranged to coincide with ultrasound pregnancy diagnosis (‘scanning’ in mid-pregnancy) and weaning 2016/17. These periods were selected because mid-pregnancy and weaning are critical periods when many welfare challenges can arise due to fluctuation in environmental and climatic conditions combined with physiological demands due to the sheep reproductive cycle [9,10,17]. Of the 32 farms involved in the study, 30 were visited twice (at mid-pregnancy and weaning), and two farms only at once (both at weaning). Mid-pregnancy visits were not conducted on two farms because either the animals or the farmers were unavailable at the right time.

### Animals and welfare assessment protocol

This study was approved by the University of Melbourne Animal and Human Ethics Committees, ethical review numbers 1613838 and 1646392 respectively. Sixty-two farm visits were conducted, and 6,200 ewes were assessed in total. Sixty-one visits were conducted by the lead researcher (C.A.M), and one visit was conducted by the principal researcher (R.E.D) who was also trained by C.A.M. At each visit, farmers were asked to provide a random sample of 100 ewes, aged from 2 to 5 years. The sample size was selected based on a power calculation to estimate a trait’s prevalence, assuming a design prevalence of 50%, a 95% confidence interval and desired precision of ±10%. This number was supported by the AWIN sheep protocol which recommends a sample of 92 sheep when the farm size is greater than 2000 breeding ewes [20]. Sheep were managed under extensive commercial conditions, in year-round outdoor systems. The assessments were performed using a holding pen and a single-file race within the farms’ regularly-used sheep yards. At each visit, we recorded the prevalence of adequate/inadequate body condition according to [21], adequate/inadequate fleece condition [20], prevalence and severity of skin lesions, adequate/inadequate tail length according to [20], low/high dag score [22] and lameness [20]. The assessment criteria of the welfare indicators are reported in Table 1. These indicators were selected because they proved to be valid, reliable and feasible for extensive sheep farming conditions [17,23], and they address main welfare concerns for sheep, covering freedom from hunger, pain, injury or disease. In addition, the number of ewes that required further care was recorded and reported back to the farmer. Further care was defined as any sick or injured ewe that would benefit from further inspection and/or intervention. This included, but was not limited, to poor body condition, poor fleece condition, severe injuries (e.g. fresh, bleeding and ≥10 cm) and severe lameness (e.g. score 2 or more). Further interventions may have included: further treatment, drafting the animal from the main flock or culling. If indicated by the farmer, the ewes classed as ‘further care’ were drafted to a holding pen for further inspection by farm staff. The course of action, or intended course of action, taken by the farmer was recorded. During the visits, farmers also had to complete a questionnaire on general information about the farm (e.g. main farming enterprise, flock size, etc) and demographic information.

**Table 1:** Animal-based welfare indicators used to assess ewe welfare.

Welfare indicators	Assessment criteria
Body condition score	Scored on a 5-point scale, using a quarter-unit precision [24,25] (1) Emaciated. Dorsal spinous and transverse processes are sharp and prominent. (2) Thin. Dorsal spinous processes are still prominent, but not as sharp. Transverse processes rounder on edges. (3) Average. Spinous and transverse processes are smoother and less prominent. (4) Fat. Considerable pressure is needed to feel dorsal spinous processes. Transverse processes cannot be felt (5) Obese. Dorsal spinous and transverse processes cannot be felt. The BCS classification was also based on recommendations from [21].
Fleece condition	Scored on a 3-point scale: (0) Good fleece condition, when parted, the fleece has no lumpiness or signs of ectoparasites (1) Some fleece loss, small shedding or bald patches ≤ 10 cm diameter. When parted, the fleece may have some lumpiness or scurf, little evidence of ectoparasites (2) Significant fleece loss with bald patches of greater than 10 cm in diameter, clear evidence of ectoparasite [20]
Skin lesions	Assessed by recording number, location and severity of the skin lesions. Lesions were classified as cuts, open wounds, old wounds or scars and abscesses.
Tail length	Scored on a 2-point scale: (0) The ventral tip of the vulva is covered by the docked tail when held down (1) The tail is over-shortened or almost not present, or if the vulva and anus cannot be covered [20]
Dag score	Scored on a 6-point scale: (0) No evidence of faecal soiling (1) Very light soiling on the breech area (2) Moderate dag on the breech area extending ventrally (3) Severe dag predominantly on the breech area, extending ventrally and dorsally over the tail some soiling and dag around the anus (4) Excessive dag on the breech area and on the hind legs (5) Very severe dag on the breech area and on the hind legs or below the level of the hocks [22]
Lameness	Scored on a 4-point scale: (0) Not lame (1) Clear shortening of stride with obvious head nodding or flicking as the affected limb touches the floor (2) Clear shortening of stride with obvious head nodding and not weight-bearing on affected limb whilst moving (3) Reluctant to stand or move [20]

### Statistical analyses

Descriptive analyses were performed in Excel (Microsoft) and in R statistical package 2.13.1 (R Development Core Team, 2008). Statistical analyses were based on prevalence data. To assess ewe welfare, the data was checked for normal distribution (Kolmogorov–Smirnov test; Q–Q, scatter and box plots) and generalised linear mixed models were conducted to investigate the associations of time of assessment (mid-pregnancy and weaning), ewe breed (meat or wool breeds) and location (high rainfall or sheep-wheat zones) on welfare outcomes, with all three and their interactions as fixed factors. In all instances, farm was set as a random factor to account for specific variation between farms. In addition, t-test was used to compared differences in flock sizes between high rainfall and wheat-sheep zones.

The results from ewes classed as ‘further care’ were grouped into quartiles, and the 25% top performing farms, that is, farms in the lower quartile for this ‘summary measure’ were identified at both time periods.

Relationships between the different welfare indicators were examined by Spearman’s Rank correlation using SAS statistical package. Correlation values were classified as moderate if between 0.3 and 0.59 or strong if ≥ 0.60. (Statistical Analysis System, Release 9.4 2012; SAS Institute Inc., Cary, NC, USA). Only those correlations significant at P ≤ 0.05 are reported.

## Results

From the 32 farms in the study, the majority of farmers were male (n = 30). The average age was 51 (ranged from 25 to 87) and the average years working with sheep was 26.7 years (ranged from 2 to 67). A total of fourteen farms (44%) were classed as meat-focused enterprises, as the primary source of income was the production of lambs for slaughter. Twelve farms (38%) were classed as dual purpose (meat-wool) enterprises, defined here as enterprises which at least 25% of income was derived each from wool and meat, and six farms (18%) were classed as wool-focused enterprises as the primary source of income was wool production. The average ewe flock size was 2,771, however, there was a wide range in flock sizes (431–9,400) and breeds used across the 32 farms which are summarised in Table 2. On average, there were significantly more ewes on farms in the high rainfall zones (n = 3,555) compared to the wheat-sheep zones (n = 1,624, *t* = 2.86, *P* = 0.008).

**Table 2:** Farm demographics according to enterprise, flock size and breed. The range of the ewe flock sizes is presented in parentheses.

Enterprise	Farms	Average flock size	Breed
Meat	14	2,770 (500–9000)	*Composite, Poll Dorset Highlander, Corriedale
Meat-wool	12	2,246 (431–4411)	Merino, Merino first-cross, Composite, Dohne
Wool	6	2,091 (1075–9400)	Merino

*Composite breeds were mainly Coopworths (Border- Romney, F3 generation progeny). Merino first-cross ewes refer to the offsprings of Merino ewes with Border Leicester rams.

### Assessment of ewes’ welfare

The results of the welfare assessment at both visits are presented in Table 3. Overall, 185 (3.0%) ewes were reported to farmers for further care. Considering both visits, there was at least one ewe reported for further care on each farm. On average, 2.6% of ewes required further care at mid-pregnancy and 3.3% of ewes required further care at weaning. The highest percentage of ewes requiring further care on a farm was 11% (n = 11) which was at weaning time. There was no influence of breed type or visit on the number of ewes that needed further care, however, location had a significant effect (F = 1.87, P = < 0.001). Ewes on wheat-sheep zones were more commonly in need of further care than ewes in high rainfall zones (n = 97 (6%) vs. n = 88 (2.5%)).

**Table 3:** Percentage, standard deviation (SD), minimum and maximum number scored in each category according to time of assessment; raw numbers presented in parentheses.

Animal Welfare Indicators	Pregnancy				Weaning				Total
	Percentage	SD	MIN	MAX	Percentage	SD	MIN	MAX	Percentage
	(n = 3,000)				(n = 3,200)				(n = 6,200)
BCS									
Thin ≤ 2.25	8.4 (253)	±9.8	0	45	12.7 (407)	±11.3	0	43	10.6 (660)
Adequate 2.5–3.5	90 (2701)	±9.6	55	100	84 (2688)	±10.1	56	100	86.9 (5389)
Fat ≥ 3.75	1.5 (46)^a^	±2.8	0	12	3.3 (105)^b^	±3.7	0	13	2.4 (151)
Fleece condition									
Score 0	91.6 (2748)	±11.6	54	100	90.3 (2891)	±16.4	23	100	91 (5639)
Score 1–2	8.4 (252)^a^	±11.6	0	46	9.7 (309)^b^	±16.4	0	77	9.0 (561)
Ewes with skin lesions	30.1 (904)	±23.5	0	94	31.5 (1011)	±28.6	1	100	30.8 (1915)
Total skin lesions	1637^a^				2624^b^				4261
Head/neck	10.8 (177)	±16.9	0	91	19.4 (508)	±50.2	0	283	16.1 (685)
Ear	35.9 (588)	±18.5	0	54	24.4 (639)	±19.2	0	64	28.8 (1227)
Eye	1.2 (19)	±1.1	0	3	0.3 (8)	±0.5	0	2	0.4 (27)
Body	17.9 (293)	±38.1	0	206	29.5 (773)	±100.1	0	560	25.0 (1066)
Rear	29.8 (488)	±45.3	0	219	19.4 (508)	±43.6	0	228	23.4 (996)
Legs	4.4 (72)	±9.7	0	53	7.0 (188)	±17.2	0	85	6.1 (260)
Dag score									
Score 0–3	98.9 (2967)	±2.2	91	100	95.4 (3054)	±12.7	31	100	97.1 (6021)
Score 4–5	1.1 (33)^a^	±2.2	0	9	4.6 (146)^b^	±12.7	0	69	2.9 (179)
Tail length									
Score 0	11.9 (356)	±16.2	0	67	16.4 (526)	±21.7	0	78	14.2 (882)
Score 1	88.1 (2645)	±16.2	15	100	83.6 (2674)	±21.7	22	100	85.8 (5318)
Lameness									
Score 0	95.7 (2872)	±3.2	85	99	94.8 (3034)	±2.6	90	99	95.3 (5909)
Score 1	2.9 (86)	±2.8	0	11	3.5 (112)	±1.8	0	7	3.2 (198)
Score 2–3	1.3 (40)	±1.0	0	4	1.7 (53)	±1.3	0	4	1.5 (93)
Further Care	2.6 (77)	±1.4	0	5	3.3 (108)	±2.3	0	11	3.0 (185)

^a,b^ Values within a row with different superscripts differ significantly at P<0.05. The BCS classification was based on recommendations from [21].

Nine farms (30%), out of the 30 farms assessed at mid-pregnancy, were classed in the lower quartile for the measure ‘further care’ (1% or fewer ewes in need of further care), and thirteen farms (40.6%), out of the 32 assessed, were classed in the lower quartile at weaning (2% or fewer ewes required further care). However, only six farms (20%), out of the 30 farms visited twice, were consistently in the lower quartile for this ‘summary measure’. In contrast, eleven farms (36%) were consistently classed in the upper quartile. At both visits, these farms had 3% or more ewes that required further care.

Some common causes of further care were moderate/severe lameness or foot-related issues, BCS of 2 or below and active dermatophilosis or broken wool. Other afflictions included severe skin lesions, active pink eye, clinical mastitis, flystrike and vulva bleeding. In 51% of the cases, ewes in compromised welfare were experiencing multiple issues. Farmers took action in 40 (20%) of the 185 cases of ewes needing further care reported. Action taken by the farmer during the researcher presence involved drafting the ewes to be checked later (n = 29) or immediately addressing the issue. Immediate actions taken were hoof trimming to correct hoof overgrowth (n = 6), draining abscesses (n = 2), treating for flystrike (n = 2) and removing ingrown horns (n = 1). Issues where no immediate action was taken include cases of moderate and severe lameness, low condition score ewes, severe injuries, active pink eye and mastitis.

Lame ewes were found on all 32 farms. A total of 68% (n = 198) of all observed lameness cases were mild (score 1) while 32% (n = 93) of all cases were moderate or severe (scores 2 and 3). Lameness was affected by visit and location (F = 5.87, *P* = 0.014) with ewes in the wheat-sheep zones having more incidents of lameness at weaning compared to mid-pregnancy.

Most ewes examined (85.7%) had short docked tails, with 2645 (88.1%) at mid-pregnancy and 2674 (83.6%) at weaning. No differences were found between breed type, visit or location. While less frequent, adequate tail lengths were present across 30 out of the 32 farms assessed and ranged between 1 to 67 ewes with adequate tail length at mid-pregnancy, and from 1 to 78 at weaning.

Most of the ewes were within the BCS range of 2.5 and 3.5. However, a total of 10.6% (n = 660) of the ewes were classed ‘thin’ (BCS of 2.25 or below). Out of these 660 ewes, 36 (5.4%) cases had a BCS of 2 or below. On the other hand, a total of 2.4% (n = 151) of the ewes were classed ‘fat’ (BCS of 3.75 or above) which were more often observed at weaning than at mid-pregnancy (F = 4.11, *P* = 0.04).

A total of 30.8% (n = 1915) of the ewes examined presented skin lesions, and the rate of lesions was higher at weaning (0.5 lesions per ewe; F = 200.46, *P* < 0.001) than at mid-pregnancy (0.29 lesions per ewe). Most common lesions were ear lesions related to sunburn (17.5%, n = 1086). Affected ears were irritated, the skin was red and presented scabs, and the extent of the lesion usually compromised 50% of the ears or more. Other common lesions were cuts to the body and rear. Eye lesions were the least with 27 ewes affected in total (0.4%). Nine of these were active pink eye and a maximum rate recorded on a farm was 2%. Skin lesions were more common in wool breeds at both mid-pregnancy and weaning visits. Five merino flocks were visited following shearing or crutching, which significantly affected the number of injuries observed per ewe (F = 1128.7, *P* < 0.001). During the visits after shearing, 86.8% of the ewes assessed (n = 434) had at least one lesion to their body, legs, rear, ears or head. The average number of skin lesions per ewe was 5.4 (range 0 to 26) and most of these lesions were to the body.

Fleece issues were observed in 9% (n = 561) of the ewes. They were more common in meat breeds than they were in wool breeds (F = 5.67, *P* = 0.02), and were more common at weaning than at mid-pregnancy (F = 5.50, *P* = 0.02). Incidents were under 5% in wool breeds, but up to 15% in meat breeds at weaning. Common fleece issues were related to fleece rot, that is, matted band of wool with either, yellow, green or brown discolouration due to bacterial growth (n = 507). Other issues were active or old lesions of dermatophilosis (n = 32) and wool loss (n = 22). Overall, only 2.9% of ewes had high dag scores (score 4 and 5), but they were found on 17 out of the 32 farms, and they were more frequently observed at weaning than at mid-pregnancy (F = 29.19, *P* < 0.001).

Table 4 shows the significant correlations observed between the different welfare indicators. At both visits, there was a moderate positive correlation between ‘fat’ ewes (BCS equal or above 3.75) and lameness. In addition to these correlations, at mid-pregnancy there was a significant negative correlation between skin lesions and ‘fat’ ewes (r = -0.67, *P* < 0.001). At weaning, there was a significant negative correlation between skin lesions and high dag scores (r = -0.44, *P* = 0.014).

**Table 4:** Spearman’s Rank correlations between the welfare indicators.

Welfare indicators		r	P-Value
**Mid-pregnancy**			
BCS ≤2.25	BCS 2.5–3.5	-0.88	< .0001
BCS ≥3.75	Skin lesions	-0.67	< .0001
	High dag scores	-0.46	0.011
	Lameness (score 1–3)	0.41	0.023
Skin Lesions	High dag scores	0.36	0.053
	Lameness (score 1–3)	-0.37	0.044
High dag scores	BCS ≥3.75	-0.46	0.011
			
**Weaning**			
BCS ≥3.75	BCS ≤2.25	-0.47	0.007
	Lameness (score 2)	0.45	0.009
Inadequate fleece	Skin lesions	0.35	0.052
Skin lesions	High dag scores	-0.44	0.011

## Discussion

Thirty-two farms in the high-rainfall and wheat-sheep zones of Victoria were involved in this study and 6,200 ewes were examined in total. To our best knowledge, this study constitutes the largest evaluation of the welfare of ewes conducted in Australia. While results in this study may be more representative of welfare problems in large-scale sheep farms, such as those in the southern hemisphere, the findings of this study are a sound basis for future research, providing valuable insight into the main welfare issues likely to be encountered in extensive sheep farming enterprises.

Overall, the welfare of the ewe flocks, based on the six indicators measured, was considered good. A total of 86.9% of the ewes were in adequate BCS, 91% had good fleece condition, 69.2% had no skin lesions, 97.1%, had low dag scores and 95.3% of the ewes were not lame. For the proportion of ewes that needed further care (3.0%), the extent of welfare compromise was significant and, in many cases, involved multiple issues. The most common causes of further care were severe lameness or foot-related issues, low body condition and active dermatophilosis or broken wool. These results are in agreement with previous on-farm studies conducted in the UK [10,26], suggesting that the welfare issues identified in this study are also relevant to some extent to sheep managed extensively worldwide. While cases of further care were identified in all farms, there were six farms (20%) that at both visits were classed in the lower quartile for the summary measure ‘further care’. These farms consistently had 2% or fewer ewes in need of further care during the assessments, performing better for this measure than the rest of the studied group. To our knowledge, this is the first study quantifying the number of ewes in need of further care on farms and we propose that a target of 2% for animals in need of further care could be a useful starting point to encourage continuous improvement for the welfare of extensively managed ewes. Future benchmarking programs could use this threshold to assess/compare farm performance and measure improvements.

In all cases of ewes classed as in need of further care, none of these cases were previously identified or treated by farm staff or the farmer, even though all ewes were recently in the yards for other husbandry procedures (e.g. ultrasound pregnancy diagnosis and weaning). We believe this highlight two issues: 1) the difficulty of identifying individual welfare compromise in large-size flocks and 2) the difficulty of decision-making about when and how to manage ewes that need further attention. While the number of ewes requiring further care was low, a limited number of ewes were immediately treated or drafted for further treatment, which does not necessarily guarantee that corrective actions were taken. Management of sick animals involves decision-making and planning, and therefore, a number of factors may be influencing farmers’ willingness to address farm issues [27]. Possible reasons for not providing immediate care, or why some cases were treated more promptly than others, may include: the issue may not have been perceived to be important by the farmer, a perceived difficulty in treating certain health issues (e.g. draining abscesses vs clinical mastitis), or time constraints. Possibly, this lack of intervention is driven by the low economic value of an individual ewe, which has been previously identified as a contributing factor to low levels of intervention [28].

A valuable and practical way that could help farmers to identify sheep at risk or in compromised welfare could be to perform close examination (‘hands-on’ inspection) of a representative sample of the flock around times where sheep are in the yards for other husbandry procedures (e.g. vaccinations, internal parasite control, weaning). Close examination of sheep should be performed more than once throughout the year to obtain accurate results as seasonal variation needs to be accounted for. Assessing welfare at critical times, such as mid-pregnancy and weaning, concentrate observations on those periods when many welfare challenges can arise [9,10,12]. In addition, providing farmers with easily accessible and practical solutions to common welfare issues could help increase the frequency with which sheep in need of further care are treated. Methods would need to be applied when ewes are in the yards or implement relatively easily in the paddock. For example, tools like decision trees and checklists that are simple to follow and readily available could be effective ways to help farmers in the management of sheep in need of further care. Another alternative could be the use of sensor technology. Currently, GPS tracking devices have been developed to assist farmers with flock monitoring and the identification of sick animals [29–31]. However, further research is required to improve the practicality of this technology in order to increase its applicability.

Lame ewes were found across all farms and while moderate and severe lameness were notified to farmers, it is difficult to assure that corrective measures were taken in all cases. Lameness was affected by visit; there was an average of 4.2% of lame ewes at mid-pregnancy and 5.2% of lame ewes at weaning. This is consistent with the normal pattern of lameness affecting sheep mostly over the winter and spring months when the soil has more moisture, predisposing sheep to footrot, foot abscess and hoof overgrowth [32,33]. In extensive sheep farming conditions, where sheep may walk on average 17 km daily [34], and there is limited monitoring of individuals, the potential for lameness to impact animal welfare is of particular concern [7]. In this study, it was not practical to always identify the cause of lameness, which was a limitation of the study. However, from an animal welfare perspective, whilst the incidence and cause of lameness are important to identify, so is how rapidly lame sheep are identified and treated. Only a few cases of mild or moderate lameness were immediately treated by the farmers of this study, and the immediate action taken was hoof trimming to correct some cases of hoof overgrowth. While treatment of lameness may be influenced by cost and labour, further studies examining other key drivers that might influence this behaviour such as time constraints, knowledge and skills of farmers are needed to develop adequate strategies to promote behavioural change and safeguard sheep welfare.

A high number of ewes (85.7%) had tails docked too short, indicating that farmers tail docking behaviour present an important risk to welfare. These results are in agreement with an interview study from South America [35] that reported that most farmers in Chile (55.9%) docked lambs’ tails shorter than recommended. Tail docking is an important management practice used to reduce the risk of flystrike. However, if the tail of the lambs is docked too short (less than the third palpable joint) sheep have a higher risk of rectal prolapse [36], flystrike [37] and bacterial arthritis [38]. Even though the negative impacts of docking tails too short have been well established, short tails were found across all farms. As tail docking is a common husbandry practice, it is possible that other classes of stock on the farms assessed also had short docked tails and the associated welfare risks. Further studies assessing different classes of sheep on farms may be valuable to clarify this issue.

Most of the ewes in this study were within recommended body condition at both mid-pregnancy and weaning, which suggests that farmer nutritional management of the whole group was good overall. However, thin ewes were observed within flocks, suggesting that some farmers in this study were not identifying/treating individual thin ewes. Maintaining ewes in adequate body condition correlates positively with most production traits, including numbers of lambs born and weaned and both, lamb and ewe survival [39]. However, ensuring that all sheep in a flock meet their nutritional requirements is not easily achievable in extensive systems [7]. While animal welfare issues can occur at both high and low body condition, thin sheep face more immediate risks to welfare generally. They have higher feeding motivation than ewes with higher BCS, have reduced ability to adapt to cold challenges [40] and are at greater risk of developing pregnancy toxaemia [41]. Body condition scoring is a simple and low-cost management practice that can help farmers to identify animals in low BCS. Further studies, however, need to assess farmers’ engagement and barriers for adoption of this practice. In addition, further research in the development of sensor technologies to identify thin and fat sheep in extensive farming systems are required to assist farmers with this issue, and therefore, mitigate the welfare compromise or risks associated with inadequate body condition.

The number and severity of skin lesions were variable, however, ear lesions due to sunburn were commonly observed. Possible reasons for these findings may be associated with lack of shelter in the paddocks and/or inadequate nutrition e.g. ingestion of toxin plants or cobalt deficiencies [42]. This suggests that other stock classes in the farms may also be affected. Further examination on nutritional management and farm resources e.g. presence, quality and type of shelter may be valuable. While body lesions were not very common, the number and severity of them increased significantly following shearing. Although only very large and open cuts were referred to the farmers, there were many instances where cuts were >5 cm in length but healed. Shearing is a stressful procedure for sheep [34], and injuries sustained during wool removal contribute significantly to this stress while also creating a risk of infection and other diseases [43]. The data collected in this study would serve as groundwork for future comparison. However, further research is needed on the short and long-term impact of shearing injuries on the welfare and productivity of extensively managed sheep.

This study’s results may not necessarily represent the welfare of ewes within the flocks of the study farms or ewes throughout Australia. While the study visits were conducted during key periods of the reproduction cycle of sheep, the population studied was robust. The ewes examined in this study were the easiest to manage and an economically valuable group in the farms; that is, single-bearing ewes (if known), from 2 to 5-year-old. Age and mortality have a parabolic relationship, with the risk being higher in younger and older animals. As a result, it is possible that the level of welfare compromise reflected on this study may be the lowest it would be across each farm. Ewes were also brought into the yards by the farmers so that the welfare assessment could be conducted. It is possible that this process may have favoured healthier animals, due to farmers bias, but also because more compromised ewes (e.g. severe lame, lethargic) are less able to move [44], and thus seriously compromised ewes were less likely to be mustered to the yards. Further on-farm research examining whole flocks and, obviously, differences between stock classes need to be conducted to validate these findings. Furthermore, although there was an adequate representation of farm enterprises and location according to the national statistics of the sheep industry [13,45]. we used voluntary recruitment, which may have introduced a participation bias into the study skewed towards more ‘proactive’ farmers or farmers more engaged with industry or research activities. Further studies could develop a nation-wide survey to collect information on demographic data, farm management and farm health records from a wide variety of farmers to broaden the reach and increase the likelihood of a more accurate representation to compare these findings.

Further refinement of the welfare assessment protocol is necessary to achieve a more comprehensive assessment. The welfare assessment of this study mainly evaluated the biological functioning of ewes, addressing important welfare issues identified by producers, industry, specialist and general public [10,14,20]. While animal-based indicators are the most important to consider, as they are direct indicators of the welfare of the animal, some relevant management- and resource-based indicators may also need to be incorporated in future assessments (such as nutrition management or provision of shelter). When combining the information gathered by animal-, management- and resource-based indicators it is possible to assess, more comprehensively, where actions need to be taken to correct or mitigate the issue or potential risks to welfare. Reduction in the number of animal-based indicators could also make this on-farm welfare assessment even more practical, and thus more likely to be implemented. Overall, there were consistent and significant associations between some welfare indicators in this study. At both visits, positive correlations were found between lameness and ‘fat’ ewes, possibly because fat sheep walk less, which predispose them to foot problems [46]. Correlations are useful in understanding associations between welfare indicators, but these associations are usually complex and not straightforward. Further examination of these relationships is necessary to determine the potential of lameness and/or BCS to act as proxy measures for a larger suite of welfare indicators. Another aspect that needs to be considered in future assessments is the evaluation of the affective state of ewes. Flight distance and qualitative behaviour assessment are two potential measures that can be used to assess emotional state in these animals [47]. How flight distance and the behaviour of sheep during routine handling may differ across farms, and how they may be associated with farmer handling behaviour and attitudes to sheep could provide further insights on the affective state of sheep and the farmer-sheep relationship in extensive systems.

## Conclusion

We conservatively conclude that the flocks assessed in this study were in good welfare, however, welfare compromise to the individual animal can be significant. The most common causes of further care were severe lameness or foot-related issues, low body condition and active dermatophilosis or broken wool. In addition, an important and prevalent risk to welfare identified across all farms was short tail length. While less prevalent, other risks to welfare such as poor fleece condition, ear lesions due to sunburn, shearing cuts and fat ewes were also identified. Overall, the issues identified in this study can arise from, and be treated or mitigated by, management practices. While results in this study may be more representative of welfare problems in large-scale sheep farms, such as those in the southern hemisphere, the findings of this study are groundwork for future research, providing valuable insight into the main welfare issues likely to be encountered in extensive sheep farming enterprises. Future studies should develop practical technologies that can assist in the detection of the welfare issues identified in this study. For example, practical GPS tracking sensors to monitor the flock and identify sheep at risk or in compromised welfare are much needed. Finally, understanding the prevalence of welfare issues is the first step to determining acceptable thresholds for the industry. Therefore, the thresholds and prevalence data identified in this study could be used for future comparison and sheep welfare benchmarking programs. For instance, a target of 2% for animals in need of further care could be a useful starting point to encourage continuous improvement in extensive sheep farming enterprises.

## Supporting information

S1 Data Supporting information. (XLSX)
